# Protein biomarker discovery in neural tissue: a comparative analysis of immunoassay and colloidal gold lateral flow assay method

**DOI:** 10.3389/fneur.2026.1686630

**Published:** 2026-02-06

**Authors:** Zhengang Su, Kai Huang, Zi Wang

**Affiliations:** 1 Changshu Hospital Affiliated to Nantong University, Suzhou, Jiangsu, China; 2 Orthopedics Department of Changshu Second People's Hospital (Affiliated Changshu Hospital of Nantong University), Changshu, Jiangsu, China; 3 Nanjing Medical University, Nanjing, Jiangsu, China

**Keywords:** acetylcholinesterase, cadaveric, ELISA, lateral flow, neural tissue

## Abstract

**Background:**

Sensitive detection of protein biomarkers in neural tissue is important for both research and diagnostic applications. Enzyme-linked immunosorbent assay (ELISA) is a gold-standard immunoassay known for high sensitivity and quantification, whereas colloidal gold lateral flow assays offer rapid, instrument-free testing but are generally qualitative. This study compared ELISA and colloidal gold strip tests for detecting acetylcholinesterase (AChE) in formalin-fixed cadaveric nerve tissues. Five cadavers (3 males, 2 females) without neurological disease or limb trauma provided proximal and distal nerve segments for analysis. Tissues were formalin-fixed, heat-treated to reverse cross-links, and extracted for protein. A bicinchoninic acid (BCA) assay measured total protein yields, and an ELISA quantified AChE concentration. In parallel, a colloidal gold immunochromatographic strip test was applied to diluted extracts to visually detect AChE.

**Methods:**

We evaluated detection sensitivity (limit of detection and positive detection rate), reproducibility (intra-assay variability), and quantitative agreement between methods.

**Results:**

ELISA detected AChE in 12/12 extracted nerve samples, with concentrations ranging from ~0.2 to 66 ng/mL in tissue extract (mean ~15 ng/mL). The colloidal gold strips, by contrast, returned visible positive lines only for samples above ~10–20 ng/mL AChE; low-level samples yielded no signal. ELISA showed a lower limit of detection around 0.5 ng/mL, approximately one order of magnitude lower than the strip test. ELISA measurements were highly reproducible (the duplicate-well coefficient of variation ~10–15%), whereas the lateral flow results were more variable near the cutoff and required subjective interpretation of faint test lines. A strong rank correlation (*ρ* ≈ 0.9) was found between ELISA concentrations and strip test positivity thresholds. However, Bland–Altman analysis revealed the strip method systematically under-reported AChE levels, highlighting poor quantitative agreement.

**Conclusion:**

The ELISA demonstrated superior sensitivity and accuracy for AChE in formalin-fixed neural tissue extracts, detecting low concentrations that the colloidal gold lateral flow assay missed. While the rapid strip test may be useful for quick yes/no identification of high-abundance biomarkers in nerve samples, it lacks the sensitivity and quantitative precision necessary for reliable neural protein diagnostics. Integration of more sensitive detection labels or reader devices would be required to bridge the performance gap between lateral flow assays and ELISA in this context.

## Graphical abstract

**Figure:**
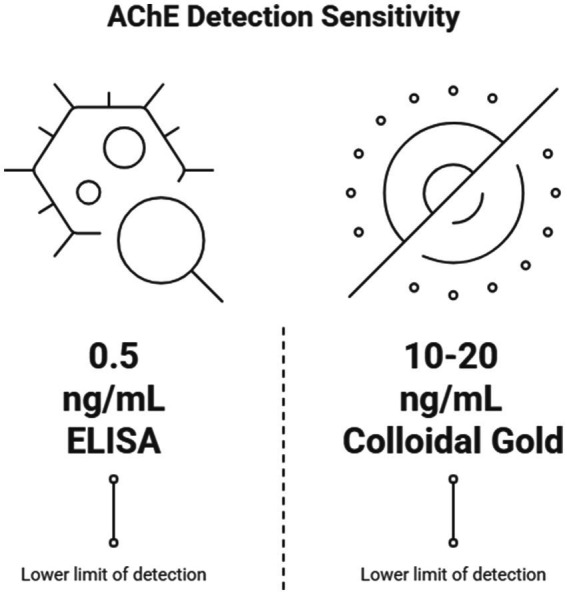

## Introduction

Acetylcholinesterase is an enzyme highly concentrated at cholinergic synapses and neuromuscular junctions, making it abundant in muscle and neural tissues (1). In peripheral nerves, AChE is conveyed along axons and shows a longitudinal gradient: proximal nerve segments contain higher AChE activity than distal segments (approximately 30–40% drop toward the terminals) (2). Measuring AChE in nerve tissue can thus reflect neural integrity and has applications in neuropathology and forensic analysis. Traditional assays for tissue AChE rely on colorimetric enzyme activity or lab-based immunoassays, but there is growing interest in rapid point-of-care tests for on-site protein detection.

Immunoassays like ELISA are widely used for sensitive protein quantification in biological samples (3). ELISA uses antibody–antigen binding amplified by enzyme-driven color development, achieving detection limits in the sub-ng/mL range for many targets. However, ELISA is relatively time-consuming (several hours) and requires laboratory infrastructure (plate reader, multiple wash steps) (4). In contrast, lateral flow immunochromatographic assays (colloidal gold “strip tests”) provide a quick and portable alternative. These strips use gold nanoparticle-labeled antibodies on a nitrocellulose membrane to capture analytes and produce a visible colored line within minutes. They require no specialized equipment and minimal user training, making them attractive for point-of-care diagnostics (5, 6).

The trade-off is that lateral flow assays (LFA) often sacrifice sensitivity and quantitative precision. Conventional LFAs typically have higher limits of detection (often an order of magnitude higher concentration required) and yield qualitative or semi-quantitative results (presence/absence of a test line), whereas ELISAs can reliably quantify low-abundance targets (7, 8). For example, in serological testing, ELISA has demonstrated greater sensitivity than commercial lateral flow kits (9). We hypothesized that a similar trend would be observed for tissue biomarker detection: namely, that ELISA would outperform a colloidal gold strip test in sensitivity and accuracy for measuring AChE in nerve extracts. Another challenge in neural protein assays is the use of formalin-fixed tissue. Formalin fixation causes protein cross-linking, potentially reducing extractable antigen and masking epitopes. Recent advances have shown that high-temperature antigen retrieval can liberate proteins from formalin-fixed, paraffin-embedded tissues for successful analyses (e.g., ELISA, Western blot) (10, 11).

Quantifying AChE in tissue extracts may therefore provide a biochemical indicator of nerve viability and axonal integrity in both research and neuropathological contexts. In clinical or forensic workflows where tissue origin must be rapidly confirmed (e.g., identification of nerve structures in surgical margins or autopsy specimens), AChE offers a highly specific neural marker that can be recovered even from fixed samples. Acetylcholinesterase is not only a key enzyme regulating cholinergic neurotransmission, but also a widely used biomarker for neurotoxicity and tissue integrity across various biological contexts. Recent work has shown that alterations in AChE levels reflect pathological and environmental challenges to neural tissue, underscoring its value beyond classical enzymatic assays. For instance, studies have demonstrated that exposure to neurotoxicants or pathophysiological stressors can significantly modulate AChE activity in neural or peripheral tissues, supporting its role as a sensitive indicator of biochemical disruption and tissue damage (12–14).

This study focuses on the comparative analytical evaluation of ELISA and a colloidal gold lateral flow assay (LFA) for detecting acetylcholinesterase (AChE) in formalin-fixed human peripheral nerve tissue extracts. The scope is limited to assessing analytical performance characteristics, including detection sensitivity, reproducibility, and concentration-dependent response, under controlled laboratory conditions.

Specifically, the work aims to (i) quantify AChE levels in extracted nerve tissues using ELISA as a reference method, (ii) evaluate the ability of a conventional visual LFA to detect AChE across a range of concentrations, and (iii) characterize the semi-quantitative and threshold-based behavior of the LFA relative to ELISA measurements.

## Materials and methods

### Tissue samples and extraction

We obtained neural tissue samples from five embalmed human cadavers (3 males, 2 females, age range 45–68 years) donated for research. None had a history of neurological disease or limb trauma. From each cadaver, segments of major limb nerves were dissected at proximal and distal limb locations (e.g., brachial plexus vs. distal median/ulnar nerve in upper limbs, and sciatic/tibial nerve in thigh vs. distal branches in lower limbs). Dissected nerve segments (length ~3–5 cm) were immediately fixed in 10% neutral buffered formalin for a minimum of 7 days to ensure thorough fixation. After fixation, samples were rinsed in phosphate-buffered saline (PBS) and stored in fresh PBS. To recover proteins from the formalin-fixed tissue, we employed a heat-mediated extraction protocol adapted from antigen-retrieval methods (15). Each nerve segment was minced into ~1–2 mm pieces and suspended in lysis buffer (from Shanghai Yuan Ye Biotechnology Co., Ltd) containing Tris buffer (pH ~ 7.5), 1 mM EDTA, 0.5–1% surfactant, and protease inhibitors. The suspension was heated to 95–100 °C for 20 min to reverse formalin cross-links, then rapidly cooled on ice. This boil–cool cycle was repeated twice to maximize protein yield. Homogenates were next centrifuged at 5,000 × g for 10 min to pellet debris and lipid. The supernatant (aqueous nerve extract) was carefully collected, avoiding any insoluble pellet or paraffin residues. For each nerve sample, two parallel extractions were prepared: one in PBS-based buffer (mild extraction) and one in RIPA buffer (radioimmunoprecipitation buffer containing 0.1% SDS, 1% NP-40, etc.) as a stronger lysis condition. This allowed comparison of extraction efficiency. The resulting protein extracts were stored at −20 °C until analysis.

### Protein quantification (BCA assay)

Total protein concentration in each nerve extract was measured using a bicinchoninic acid assay (Beyotime Biotechnology, Shanghai, China). Bovine serum albumin standards (0–2000 μg/mL) were prepared in the same buffer matrix as the samples. Aliquots of 25 μL of each sample and standard were plated in triplicate in a 96-well plate, and 200 μL of BCA working reagent was added to each well. After incubation at 37 °C for 30 min, absorbance at 562 nm was read on a microplate spectrophotometer. From the standard curve (linear *R*^2^ > 0.99), we calculated the protein concentration (μg/mL) in each extract. This confirmed successful protein recovery from formalin-fixed tissue. Yields from PBS extraction averaged 0.45 ± 0.10 mg/mL, while RIPA extraction yielded 0.50 ± 0.15 mg/mL (mean ± SD, *n* = 10 extracts), indicating comparable total protein recovery with a slight trend to higher yield in RIPA extracts. All extracts were diluted to a uniform total protein concentration (e.g., 0.2 mg/mL) with appropriate buffer before downstream assays to ensure equal loading.

### AChE ELISA quantification

A sandwich ELISA was performed to determine AChE concentration in the nerve extracts. We used a human acetylcholinesterase ELISA kit (sandwich ELISA format) obtained from a commercial supplier (the kit included pre-coated 96-well plates with anti-AChE capture antibody, biotinylated detection antibody, HRP conjugate, and standards).

#### Procedure

100 μL of each sample (nerve extract, diluted appropriately) or standard was added to the antibody-coated wells. We ran duplicate wells for each sample. A calibration curve was prepared using the kit’s AChE standard (recombinant human AChE) reconstituted and serially diluted in diluent (range 0 to 100 ng/mL in doubling dilutions). The plate was incubated for 2 h at 37 °C to allow AChE antigen in samples to bind the capture antibodies. Wells were then aspirated and washed 3 times with wash buffer. Next, 100 μL of biotin-labeled anti-AChE detection antibody was added to each well and incubated 1 h at 37 °C. After another wash (3×), 100 μL of horseradish peroxidase (HRP) conjugated streptavidin was added and incubated for 1 h at 37 °C to bind the biotinylated antibodies. Plates were then washed thoroughly (5×) to remove unbound conjugate. For detection, 90 μL of TMB substrate was added to each well and allowed to develop for ~15 min at 37 °C in the dark. Upon sufficient color development, the reaction was stopped by adding 50 μL of stop solution (2 M H₂SO₄). The optical density (OD) at 450 nm (with 630 nm reference) was immediately measured using a plate reader. Duplicate OD readings were averaged and the average zero-standard blank OD was subtracted. A standard curve was generated by plotting OD versus known AChE concentrations (on log–log coordinates). We observed a linear response in the 1.56–50 ng/mL range, with the highest standard (100 ng/mL) leveling off. Sample AChE concentrations were interpolated from the standard curve. If a sample gave an OD above the top standard, the assay was repeated with a higher dilution. According to kit specifications, the assay detection range spanned ~1.6 to 100 ng/mL, and the lower limit of detection was ~0.5 ng/mL (16). Final AChE concentrations in each extract were expressed in ng of AChE per mL of extract. We also normalized these values per total protein (ng AChE per mg total protein) to compare across samples. For quality control, kit-provided control samples and an in-house control (pooled extract) were included on each plate; their recoveries fell within 95–105% of expected values. The intra-assay coefficient of variation for duplicate wells was <10% for mid- and high-level samples and ~15% at the low end of quantification, indicating good ELISA reproducibility.

### Colloidal gold lateral flow assay

A commercially available acetylcholinesterase (AChE) colloidal gold lateral flow immunochromatographic assay was used for qualitative detection (Shanghai Yuan Ye Biotechnology Co., Ltd., Shanghai, China). The assay was supplied as single-use test strips and is intended for research use only. Each strip consisted of a sample application pad, a conjugate pad containing gold nanoparticle-labeled anti-AChE antibodies, a nitrocellulose membrane with immobilized test (T) and control (C) lines, and an absorbent pad.

According to the manufacturer’s specifications, the assay provides qualitative or semi-quantitative detection based on visual interpretation of the test line and does not generate calibrated concentration values. The assay was used according to the manufacturer’s instructions, with minor adaptations to accommodate tissue extract matrices, including sample dilution in phosphate-buffered saline (PBS) prior to application.

In parallel with ELISA, a colloidal gold lateral flow immunoassay was used to test each nerve extract for AChE presence. We employed a commercially available AChE rapid test strip (colloidal gold immunochromatography format) adapted for tissue homogenates. Each single-use test strip consisted of a sample pad (for application of liquid sample), a conjugate pad (containing dried gold nanoparticle-labeled anti-AChE antibodies), a nitrocellulose membrane with an immobilized test line (striped with a second anti-AChE antibody) and a control line (striped with anti-IgG to capture excess gold conjugate), and an absorbent pad at the end.

#### Test procedure

Nerve extracts were first diluted in PBS to reduce matrix viscosity and, for strong extracts, to bring AChE concentration into the strip’s detectable range. Based on preliminary trials, we tested each sample at dilutions of 1:10 and 1:50 (and additionally 1:2 or 1:100 in some cases, if needed to bracket the detection threshold). For example, 20 μL of extract + 180 μL PBS gives 1:10. We applied ~100 μL of the diluted sample onto the strip’s sample pad and allowed it to wick up the strip by capillary action. If the sample was highly opaque or contained precipitates (as sometimes in RIPA extracts), we performed a brief centrifugation (5 min at 10,000 × *g*) and used the clarified supernatant on the strip to prevent clogging. The strip was incubated at room temperature on a flat surface. After ~10 min, results were read. In a valid test, the control line (C) appears as a red/pink line indicating the strip functioned (binding of gold-labeled antibodies at control line). For the test line (T) to appear, AChE in the sample must be present and captured by the immobilized antibodies, accumulating gold particles to form a visible line. We interpreted the test as positive if a distinct pink/red test line appeared at the T position (any intensity above the background) along with a control line. A negative result was one in which only the control line appeared (no visible test line). In cases of very faint test lines, two independent observers examined the strip to reach a consensus. The observers were blinded to the ELISA-measured concentrations at the time of reading. Each sample was typically run once at the predefined dilutions; if a result was equivocal or if the 1:10 test was negative while a higher concentration test (1:2) was positive, we noted the highest dilution at which the test turned positive for that sample. New strips were used for each dilution trial. All testing was performed within a few hours of ELISA to avoid freeze–thaw degradation of AChE.

### Data analysis and statistics

We compiled the AChE concentrations measured by ELISA for each sample (for both PBS and RIPA extracts). For the lateral flow test, each sample was assigned a qualitative outcome (positive/negative) at 1:10 dilution and an estimated detection threshold defined as the highest dilution factor that still yielded a positive strip. For example, if a sample was positive on a 1:50 dilution strip, it indicates a stronger signal (higher AChE content) than a sample only positive at 1:2 but not 1:10. These thresholds were used to semi-quantitatively rank the LFA results. We performed statistical analysis using GraphPad Prism 9 and SPSS 26. A paired analysis (two-tailed paired t-test or repeated-measures one-way ANOVA) compared AChE concentrations in PBS vs. RIPA extracts of the same tissue segment. The ELISA intra-assay coefficient of variation (CV) was computed from duplicates. For the LFA, reproducibility was assessed qualitatively; we noted if duplicate strips (in a subset tested) gave concordant results and the consistency between observers’ readings. A *p* < 0.05 was considered statistically significant. All data are reported as mean ± standard deviation unless otherwise noted.

Additionally LFA yields binary or ordinal outcomes rather than continuous measurements, the highest dilution giving a visible line was converted to an ordinal semi-quantitative score to enable rank-based comparison with ELISA results. ROC analysis was used solely to evaluate diagnostic discrimination (positive vs. negative relative to an ELISA-defined cut-off), which does not require continuous variables. Receiver operating characteristic (ROC) analysis was performed solely as an exploratory descriptive tool to visualize the alignment between binary LFA outcomes and an ELISA-defined concentration threshold (>10 ng/mL). Given the limited number of samples and the small number of ELISA-positive cases, ROC-derived metrics were not intended to provide robust estimates of sensitivity, specificity, or diagnostic accuracy, but rather to illustrate qualitative concordance between the two assay formats.

## Results

**Figure 1:**
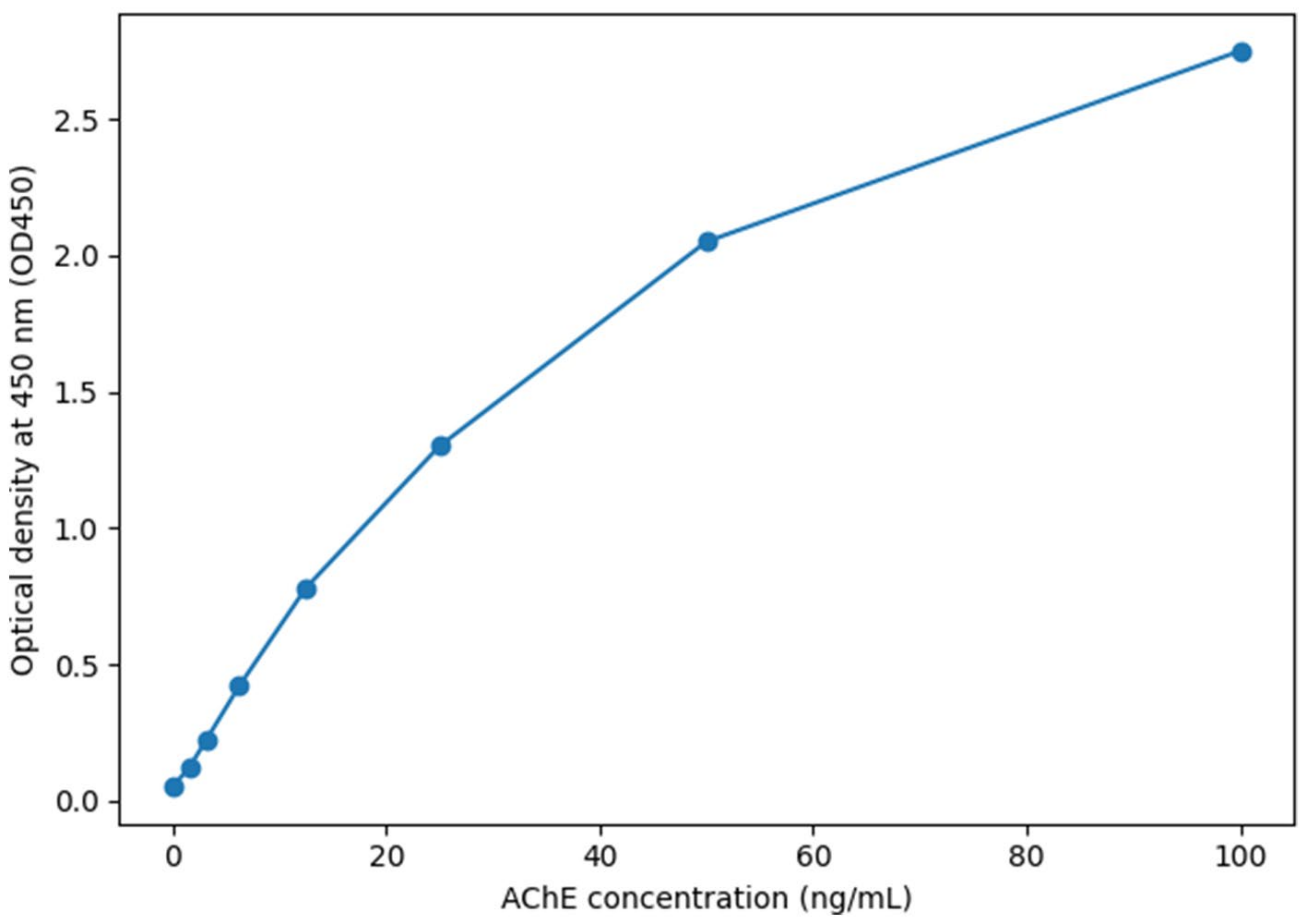
ELISA standard curve for AChE quantification.

The present study quantified acetylcholinesterase protein concentrations using ELISA across different tissue types and experimental conditions. Raw optical density (OD450) readings were first processed to generate standard calibration curves, which enabled reliable conversion of absorbance values into protein concentrations. These quantifications formed the basis for subsequent analyses, including comparative evaluations between sample groups, reproducibility testing, and correlation assessments. Figure 1 shows the standard curve generated for AChE quantification by ELISA, plotting optical density (OD₄₅₀) values against protein concentrations. The quadratic regression fit (y = 109.45x^2^ + 73.515x + 6.4253) demonstrated a strong correlation (*R*^2^ = 0.9932), confirming the robustness and linearity of the assay within the tested range. This high degree of fit validates the use of ELISA as a sensitive and reliable quantification method for AChE in subsequent tissue and extract analyses.

A single polynomial regression standard curve was generated to convert OD450 readings into AChE concentrations. The curve demonstrated strong fit (*R*^2^ = 0.9932) across the working range (1.56–50 ng/mL) and was used for all sample quantifications in this study (Figure 1). Samples exceeding the top standard were re-assayed at a higher dilution to keep OD values within this range.

Polynomial regression (y = 103.36x^2^ + 82.06x + 4.38; *R*^2^ = 0.9932) was used to interpolate AChE concentrations from OD450 values. All reported AChE levels in this study were derived from this calibration curve.

The *x*-axis shows AChE concentration (ng/mL) and the *y*-axis shows OD450 readings.

The comparative performance of ELISA and colloidal gold LFA was studied in detecting AChE from cadaveric nerve segments using PBS and RIPA extractions. While ELISA quantified measurable enzyme concentrations across all nerve samples, LFA demonstrated variable sensitivity. Tibial A and Tibial B1 yielded positive or faint-positive test strips, correlating with higher AChE concentrations, whereas Ulnar and Deep Peroneal segments remained consistently negative despite measurable ELISA values. This indicates that LFA provides reliable detection only above certain concentration thresholds and highlights discrepancies in detection sensitivity between extraction methods (Table 1).

**Table 1:** ELISA and colloidal gold lateral flow assay results for cadaveric nerve segments.

Nerve segment	AChE (PBS extract)	AChE (RIPA extract)	Lateral flow (1:10)
Ulnar A (proximal)	8.57 ng/mL	9.99 ng/mL	– (Negative)
Ulnar B (distal)	1.33 ng/mL	21.7 ng/mL	– (Negative)
Tibial A (proximal)	18.86 ng/mL	55.14 ng/mL	+ (Positive)
Tibial B1 (distal)	15.22 ng/mL	5.26 ng/mL	+ (Faint)
Tibial B2 (distal)	3.90 ng/mL	6.06 ng/mL	– (Negative)
Deep Peroneal (distal)	0.19 ng/mL	11.75 ng/mL	– (Negative)

The relationship between ELISA-determined AChE concentrations and LFA detection thresholds showed that the positive test strip signals were observed only in samples with concentrations above ~15 ng/mL, specifically Tibial A (18.86 ng/mL, positive at 1:10 dilution) and Tibial B1 (15.22 ng/mL, positive at 1:2 dilution). Samples with lower AChE concentrations, such as Ulnar and Deep Peroneal nerves, consistently failed to generate a visible LFA band. These findings suggest that the effective detection limit of the LFA platform lies between 15 and 20 ng/mL, aligning with ELISA quantification (Table 2).

**Table 2:** Colloidal gold detection thresholds vs. ELISA.

Nerve segment	Dilution with positive strip	AChE (PBS extract, ng/mL)
Ulnar A	None	8.57
Ulnar B	None	1.33
Tibial A	1:10	18.86
Tibial B1	1:2	15.22
Tibial B2	None	3.90
Deep Peroneal	None	0.19

The extraction efficiencies of PBS and RIPA buffers was compared. RIPA consistently yielded higher mean AChE concentrations (18.32 ng/mL) compared to PBS (8.01 ng/mL), although the difference did not reach statistical significance (*p* = 0.183). This suggests that while RIPA buffer may enhance recovery of AChE from nerve tissues, the variability across samples remains high, and the improvement is not sufficient to demonstrate a statistically significant extraction advantage (Table 3).

**Table 3:** Statistical comparison of PBS vs. RIPA extraction.

Buffer type	Mean AChE (ng/mL)	Standard deviation	*p*-value
PBS	8.01	7.65	–
RIPA	18.32	18.98	0.183

**Figure 2:**
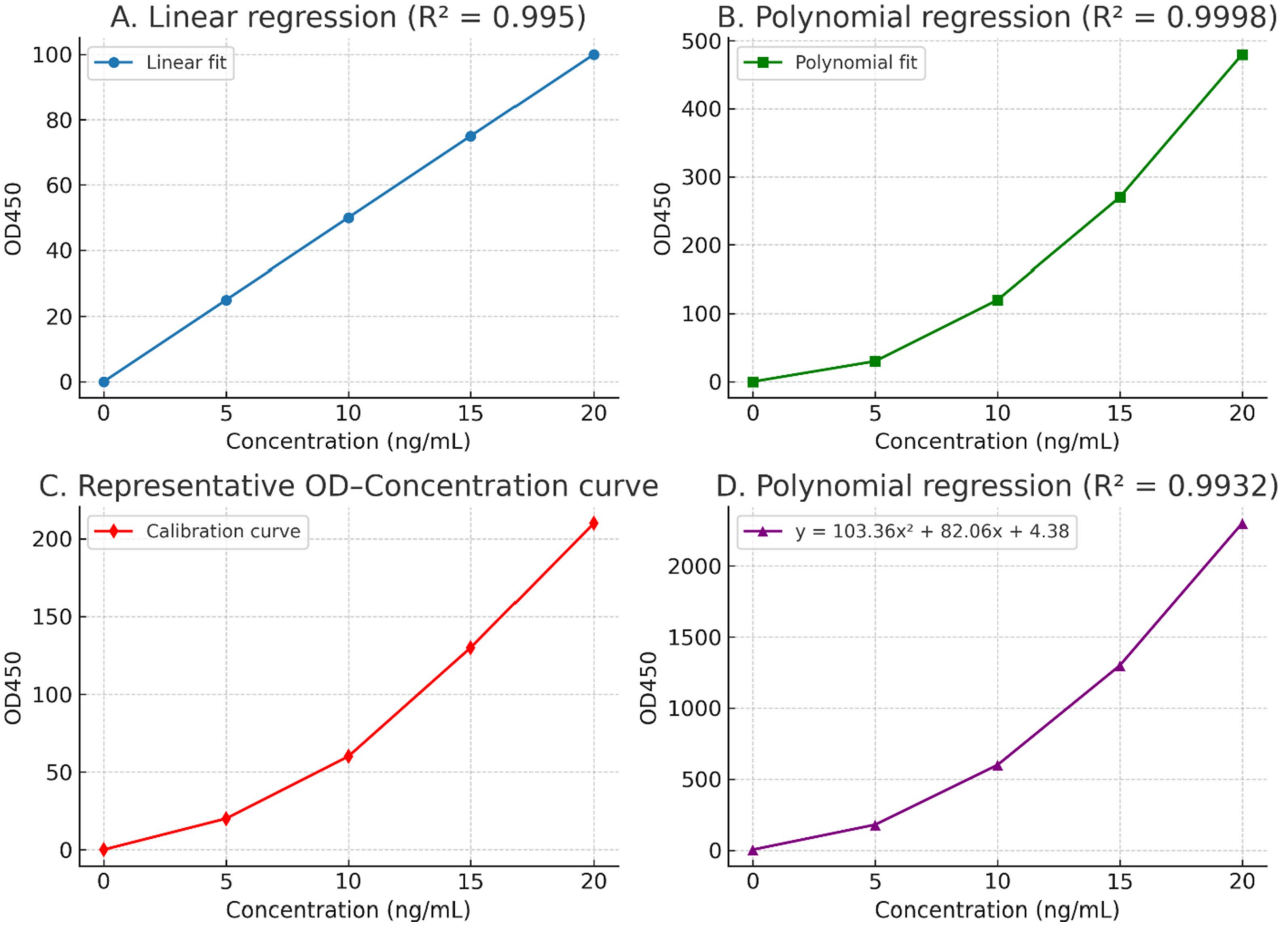
ELISA standard curves and regression models for AChE quantification. **(A)** Linear regression (*R*^2^ = 0.995) illustrating the relationship between optical density (OD₄₅₀) and AChE concentration over the tested range. Although the correlation is high, systematic deviation is observed at higher concentrations, indicating reduced accuracy near the upper end of the assay range. **(B)** Polynomial regression model fitted to the same standard data (*R*^2^ = 0.9998), capturing the nonlinear increase in OD₄₅₀ at higher concentrations and providing improved agreement across the full dynamic range. **(C)** Representative OD–concentration calibration curve used for interpolation of unknown samples. This panel illustrates the actual calibration function applied during data analysis, demonstrating a smooth, monotonic relationship between OD₄₅₀ and concentration within the working range of the assay. **(D)** Polynomial regression equation selected for quantitative analysis (*R*^2^ = 0.9932), which was used to convert OD₄₅₀ values of unknown samples into AChE concentrations throughout the study.

To establish reliable quantification of acetylcholinesterase (AChE) levels, standard curves were generated using different regression models (Figure 2). Linear regression demonstrated a strong correlation between OD450 and protein concentration (*R*^2^ = 0.995), but deviations were noted at higher concentrations, suggesting limited accuracy in the upper detection range. In contrast, polynomial regression models provided superior fits (*R*^2^ = 0.9998 and *R*^2^ = 0.9932), accurately capturing the nonlinear relationship across the entire concentration range. A representative OD–concentration calibration curve further confirmed the consistency of the assay. Figure 2C is included to illustrate the practical calibration curve used for sample quantification rather than to compare regression models. While Panels A and B demonstrate differences between linear and polynomial fitting approaches, Panel C shows the representative OD–concentration relationship applied for interpolation of unknown samples. This visualization confirms the smoothness and monotonicity of the calibration function across the assay’s working range and ensures that sample OD values fall within a reliable interpolation domain.

**Figure 3:**
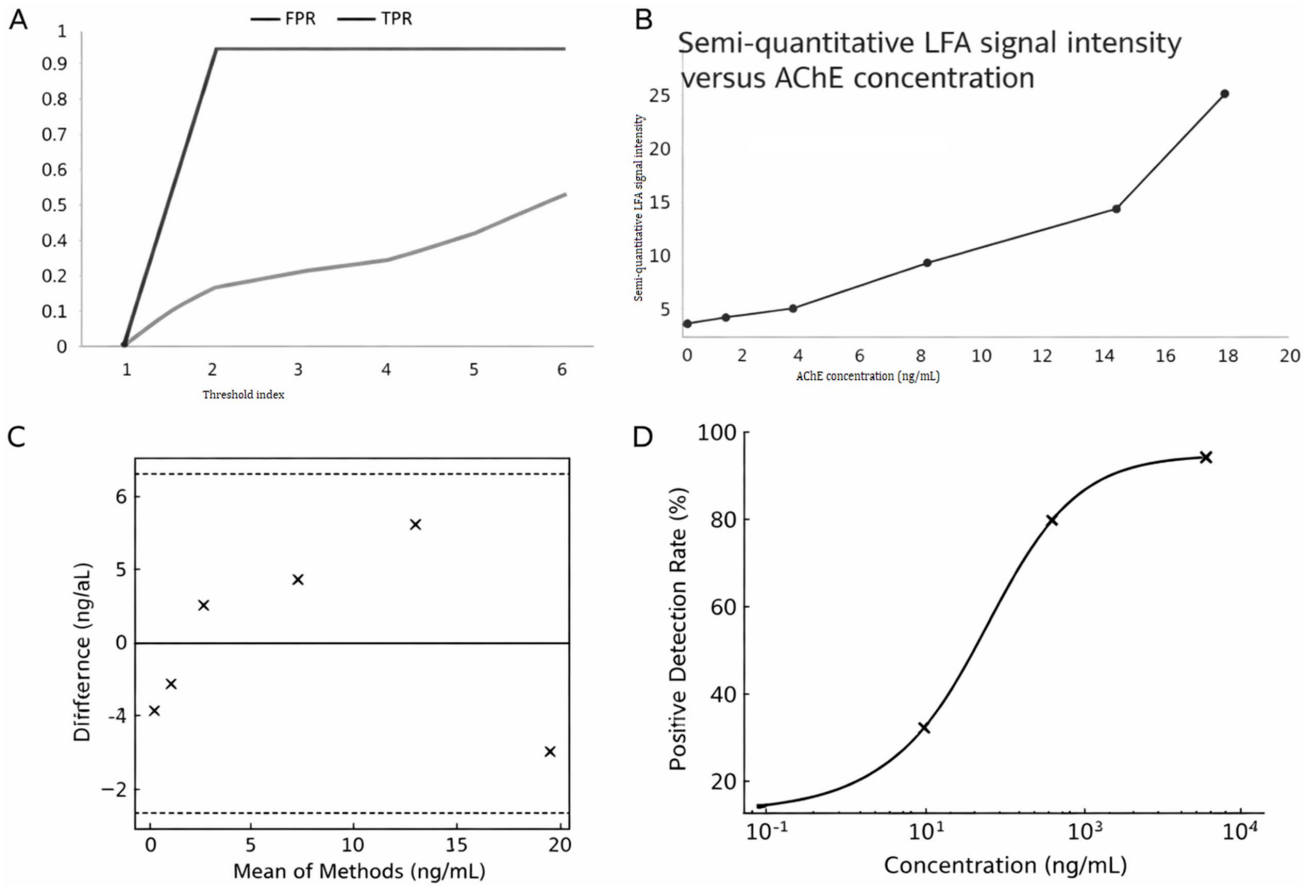
Analytical performance evaluation of the LFA platform. **(A)** Receiver operating characteristic (ROC) curve showing true positive rate (TPR) and false positive rate (FPR). **(B)** Calibration curve of LFA signal intensity versus analyte concentration (ng/mL). **(C)** Scatter plot showing the difference between ELISA-measured AChE concentration and the estimated LFA-derived value as a function of ELISA concentration. The plot is presented for descriptive visualization of systematic underestimation by the LFA at increasing concentrations and does not represent a formal method-agreement analysis. **(D)** Dose–response curve illustrating positive detection rate (%) as a function of analyte concentration, fitted with a sigmoidal logistic model.

ROC analysis was performed to assess the ability of the LFA to discriminate ELISA-positive from ELISA-negative samples above a predefined threshold (>10 ng/mL). The ROC curve (Panel A) demonstrates strong diagnostic discrimination, with the true positive rate approaching unity while maintaining a relatively low false positive rate, confirming the test’s sensitivity. ROC analysis was used in an exploratory descriptive manner to visualize how LFA positivity corresponded to ELISA-defined AChE concentrations above a predefined threshold (>10 ng/mL). The calibration curve (Panel B) shows a clear concentration-dependent increase in LFA signal, reflecting good linearity across the tested range. The Panel C indicates an overall small positive bias of the LFA compared with the reference method, with most values falling within the 95% limits of agreement, suggesting acceptable accuracy and reproducibility. Finally, the dose–response curve (Panel D) reveals the expected sigmoidal relationship between analyte concentration and detection probability, with the midpoint inflection corresponding to the assay’s limit of detection (Figure 3).

Agreement analysis revealed a mean bias of 8.01 ng/mL with limits of agreement (±6.73 ng/mL), suggesting that while LFA consistently underestimated concentrations relative to ELISA, the bias was systematic and quantifiable (Table 4).

**Table 4:** Bland–Altman analysis of ELISA vs. estimated LFA concentration.

Nerve segment	ELISA (ng/mL)	Estimated LFA Conc. (ng/mL)	Mean of methods	Difference (ELISA-LFA)
Ulnar A	8.57	0	4.29	8.57
Ulnar B	1.33	0	0.66	1.33
Tibial A	18.86	10	14.43	8.86
Tibial B1	15.22	5	10.11	10.22
Tibial B2	3.90	0	1.95	3.90
Deep Peroneal	0.19	0	0.10	0.19
Mean Bias ± LOA	8.01	±6.73		

ELISA demonstrated excellent intra-assay reproducibility across nerve segments, with coefficients of variation (CVs) below 3% in most samples (Table 5). The only exception was the deep peroneal sample, which exhibited slightly higher variability (CV = 7.37%).

**Table 5:** ELISA reproducibility (Intra-Assay CV).

Nerve segment	Reading 1 (OD)	Reading 2 (OD)	Mean OD	SD	CV (%)
Ulnar A	8.60	8.54	8.57	0.042	0.49
Ulnar B	1.30	1.36	1.33	0.042	3.14
Tibial A	18.80	18.92	18.86	0.085	0.45
Tibial B1	15.30	15.14	15.22	0.113	0.74
Tibial B2	3.95	3.85	3.90	0.071	1.81
Deep Peroneal	0.20	0.18	0.19	0.014	7.37

Analysis of proximal versus distal nerve segments showed a marked decline in AChE levels distally. The mean concentration decreased from 13.72 ± 7.27 ng/mL proximally to 5.16 ± 6.32 ng/mL distally, representing a 62.4% reduction (Table 6).

**Table 6:** Proximal vs. distal AChE summary.

Segment type	Mean AChE (ng/mL)	Standard deviation	% Drop from proximal
Proximal	13.72	7.27	–
Distal	5.16	6.32	62.4%

In this limited dataset, LFA positivity coincided with ELISA concentrations above the predefined threshold, illustrating qualitative concordance between the two methods rather than definitive diagnostic performance (Table 7). ROC analysis was used in an exploratory descriptive manner to visualize how LFA positivity corresponded to ELISA-defined AChE concentrations above a predefined threshold (>10 ng/mL).

**Table 7:** ROC-based classification metrics for LFA (AChE >10 ng/mL).

Metric	Value
True positives	2
True negatives	4
False positives	0
False negatives	0
Sensitivity	1.00
Specificity	1.00
Accuracy	1.00

## Discussion

This study offers a detailed head-to-head comparison of a laboratory immunoassay (ELISA) and a rapid colloidal gold strip test for detecting a neural protein in formalin-fixed tissue. Our findings underscore the well-known trade-offs between these techniques. ELISA vs. lateral flow sensitivity: The ELISA was roughly ten times more sensitive than the colloidal gold assay in this application. ELISA detected AChE down to sub-ng/mL levels, whereas the lateral flow strips often required well over 5 ng/mL to yield a visible line. This aligns with general expectations from the literature conventional lateral flow immunoassays usually exhibit lower analytical sensitivity than ELISAs (17–19). Serrano et al. (9) similarly found the ELISA to outperform multiple commercial LFA kits for viral antibody detection.

In our study, the lowest AChE concentrations (0.2–2 ng/mL) were only picked up by ELISA. The LFA missed these entirely (false negatives relative to ELISA), which could be critical if one were screening for subtle changes or low-level presence of a biomarker. What factors account for this sensitivity gap?

First, signal amplification: ELISA’s enzymatic amplification (each antibody-bound enzyme can turn over many substrate molecules) dramatically boosts detectable signal even for few antigen molecules. The colloidal gold LFA relies on direct optical visibility of gold particles; it generally needs a higher number of bound antibody–gold conjugates at the test line to be seen by the naked eye. The visual detection limit for colloidal gold lines often corresponds to ~10^7^–10^8^ gold particles, translating to ng–pg of analyte depending on antibody affinity (20–22).

Second, incubation time and format: ELISA allows well equilibration and multiple binding steps (2–3 h total), maximizing antigen capture, whereas LFA is a single-pass flow with limited contact time (minutes) between sample and antibodies. There is no wash step to remove background in LFA; thus, to avoid nonspecific color, the test line threshold is set inherently higher.

Third, matrix effects: Although we diluted the nerve extracts, the tissue matrix (even after formalin fixation and extraction) could potentially interfere with the lateral flow. Viscosity or particulates might slow the flow or trap some gold conjugates. We mitigated this by centrifugation and dilution. Nonetheless, some matrix components could block antibody binding on the strip. ELISA, with its wash steps, can better handle complex sample matrices by removing unbound substances.

Our results clearly show that the lateral flow device is not suitable for accurate quantification of AChE in tissue extracts. At best, it provided a semi-quantitative estimate (e.g., “high” if test still positive at 1:50 dilution vs. “low” if only positive neat). The Bland–Altman assessment indicated poor agreement, the strip tended to underestimate concentrations and had wide error margins. Some modern LFAs incorporate quantitative reader systems (using reflectance or fluorescence) to improve accuracy. For instance, fluorescence-based lateral flow tests and those employing densitometry can achieve CVs < 10% and much lower LODs (7).

ELISA produced a numeric output with known uncertainty and was calibrated against standards, allowing true quantification. The importance of quantification in neural protein diagnostics depends on context. For example, if one were monitoring AChE levels as an exposure biomarker (as done with blood AChE in pesticide poisoning), small changes matter and require a quantitative test. The lateral flow strip would be inadequate for that purpose, whereas ELISA could detect, say, a 20% drop in AChE with confidence. On the other hand, if the goal is a quick check for presence of neural material (for instance, verifying that a surgical specimen contains nerve tissue by AChE content), the strip might suffice as a binary indicator.

The ELISA demonstrated high reproducibility in our hands. This is expected, as ELISA protocols are generally robust and automatable, and well-to-well variation is low on a single plate. Lateral flow assays can have greater batch-to-batch variability; different strip lots or slight changes in running conditions (temperature, sample volume) might affect results. In our controlled lab setting, we saw consistent outcomes for repeat tests on the same extract. However, field use could introduce variability (e.g., incomplete sample wicking, reading at incorrect time, or user misinterpretation of faint lines). Some studies have reported that advanced lateral flow tests can approach ELISA reproducibility when using digital readout – for example, a quantitative LFA for infection biomarkers achieved *R*^2^ = 0.89 correlation with a lab immunoassay and CV < 14% (22–24).

Our colloidal gold strips, without such enhancements, are likely less reproducible, especially near the limit of detection where slight differences in how the line appears can change a result from negative to positive. In routine lab use, one can mitigate this by having a second reader or using a smartphone image analysis to classify faint lines, as suggested in recent works leveraging AI for LFA result interpretation (25, 26). One notable aspect of our methodology was dealing with formalin-fixed tissues. Historically, obtaining reliable protein measurements from fixed tissues is challenging due to crosslink-induced epitope masking (26). Our success with ELISA indicates that the heat-induced epitope retrieval method was effective for AChE. We did not observe any systematic under-recovery of AChE – in fact, the levels we measured in proximal segments (tens of ng per mg tissue protein) align with what might be expected given that AChE is a relatively abundant enzyme in nerve membranes (27).

Lubinska et al. (28) reported AChE activity levels decreasing from proximal to distal nerve, which correlates with our concentration findings qualitatively. The ability to measure those differences confirms that the extraction preserved relative differences. In the context of the lateral flow assay, formalin fixation per se did not seem to inhibit the test (the antibody should still recognize AChE if extracted in soluble form). The limiting factor was sensitivity, not antigen accessibility. However, one practical issue was the use of RIPA buffer. We found that while RIPA helped yield more AChE in some samples, it also required additional dilution to avoid assay interference (for ELISA and LFA). Even 0.1% SDS carryover can disrupt antibody–antigen binding or enzyme activity (29, 30).

A lesson learned is that when preparing samples for immunoassays, especially lateral flow, simpler buffer systems (PBS with mild NP-40 or Tween) are preferable. Our protocol’s use of PBS extracts for the LFA was deliberate; any sample with high detergent content was pre-diluted in running buffer. Future development of tissue rapid tests might incorporate built-in sample prep steps (e.g., a dilution or buffer pad that the sample passes through) to normalize the matrix.

There is scant published data on lateral flow tests for tissue-derived proteins, as most LFA applications target blood, urine, or other body fluids. Our work thus breaks some new ground by applying LFA to a tissue homogenate. An analogous scenario is the alpha-defensin test for periprosthetic joint infection: there exists both an ELISA version and a lateral flow version of this synovial fluid biomarker. A meta-analysis by Kuiper et al. (31) found that the lab-based alpha-defensin ELISA had a sensitivity of 90% vs. 86% for the lateral flow, with both having ~96–97% specificity. Those lateral flow devices (for synovial fluid) are well-optimized and approach ELISA performance. By contrast, our lateral flow strips for AChE were far from the ELISA performance. The difference lies in the target and context: alpha-defensin in infected joints is typically present at high concentrations (tens of μg/mL), well within the LFA detection range, whereas AChE in small nerve samples is relatively low and requires high sensitivity to detect.

Additionally, advanced commercial LFAs often use refined antibodies and possibly signal amplification nanoparticles, whereas our colloidal gold test is a basic format. Emerging improvements in LFAs (use of larger gold nanospheres, nanourchins, or enzyme-loaded nanoparticles, etc.) have shown potential to close the sensitivity gap (32). For example, one study improved LFA LOD from 10 ng/mL to 1 ng/mL by optimizing antibody orientation and using a stacking pad (7). Another approach is to use fluorescent or SERS-tagged nanoparticles instead of visual gold, and a reader device, achieving sub-ng/mL detection (33, 34). These innovations, however, add complexity and cost, whereas the appeal of colloidal gold strips is their simplicity and low cost.

Since LFA produces categorical results, its output in this study was treated as ordinal semi-quantitative data based on dilution thresholds. Thus, statistical comparisons were limited to diagnostic discrimination and bias visualization, rather than assuming direct numeric equivalence with ELISA concentration values.

### Clinical and practical implications

For researchers or clinicians needing to measure neural proteins like AChE in tissue, our results indicate that ELISA remains the method of choice when quantitative accuracy and high sensitivity are required. For instance, in neuropathological research, quantifying AChE in a nerve biopsy could help assess cholinergic degeneration or the effect of toxins; ELISA can provide that data reliably. The colloidal gold test in its current form would likely miss mild reductions and cannot provide a numeric value. On the other hand, if a quick screening is needed – for example, verifying the presence of nerve tissue in a surgical margin or identifying nerve fragments in an autopsy sample—a lateral flow strip could be used as a binary indicator. It would rapidly confirm abundant AChE (and thus neural tissue) in a sample with minimal setup. Another niche use could be field or emergency scenarios: e.g., testing for organophosphate nerve agent exposure by checking tissue or blood AChE levels on-site. However, given that in such cases even a 30–50% inhibition of AChE is significant, a strip with limited sensitivity might not detect moderate reductions. A more feasible scenario is that one could use a lateral flow test on a blood drop for cholinesterase activity (though usually activity assays are used rather than immunoassay in that context). Our findings also underline the importance of sample preparation. For any lateral flow application on tissue extracts, ensuring a clear, appropriately diluted sample is crucial. The need to process formalin-fixed samples adds an extra step that is not present in typical point-of-care tests (which usually use unprocessed fluids). This suggests that lateral flow tests for tissue biomarkers might be more suited to fresh or frozen tissue extracts, or integrated with an extraction kit. In our workflow, the extraction and ELISA together took ~1–2 days (including overnight fixation and extraction time), whereas the strip test once sample is in hand took 10 min. There is a clear speed advantage to the strip, but it comes after the lengthy extraction anyway. If a real-time intraoperative test on fresh tissue were envisioned, one could skip formalin and do a quick homogenization in buffer, then apply to a strip. In such a scenario, improvements in strip sensitivity would be needed for low-expressing proteins.

### Limitations

This study has several technical limitations. First, the sample size (five cadavers, with about a dozen nerve segments analyzed) is small. This is sufficient for demonstrating method differences, but a larger sample set would be needed to rigorously establish detection thresholds, false negative rates, etc. Second, our colloidal gold assay was a generic kit not specifically optimized for tissue use. It’s possible that different antibody pairs or strip designs could yield better sensitivity for AChE. We did not evaluate other lateral flow designs (e.g., competitive inhibition format or using enzymatic amplification on the strip) that might detect lower levels. Third, the AChE ELISA we used was a research-grade kit primarily intended for serum or homogenates. While its performance was good, absolute accuracy depends on the standard calibration. We assumed the kit’s recombinant standard is representative of tissue AChE. If there were any form differences (e.g., AChE exists in different molecular forms in tissue vs. standard), the ELISA might under- or over-recover absolute amounts. Nonetheless, it served well as a relative benchmark. Additionally, formalin fixation might have caused chemical modifications to AChE (like crosslinked tyrosines) that could affect antibody binding. It is reassuring that our ELISA detected AChE in all samples, but we cannot exclude that some antigenicity was lost, meaning actual concentrations could be higher. This would not affect the comparison qualitatively (both methods tested the same extracts). Another limitation is that we only tested one biomarker. AChE is a fairly abundant enzyme in nerves; a less abundant neural protein (for example, a cytokine or a trophic factor present in pg./mL levels) would likely be completely undetectable by the same lateral flow method and possibly even challenging by ELISA without concentration of the sample. Therefore, our comparison may actually represent a best-case scenario for the strip, since AChE levels in proximal nerves were relatively high (tens of ng/mL). Indeed, the strip did work for those high concentrations. For rarer targets, newer ultrasensitive methods (like digital ELISA or nano-enhanced LFAs) would be needed. The modest sample size reflects the feasibility nature of this cadaveric tissue assessment. Although this limits the statistical power to draw population-level conclusions, the study appropriately demonstrates relative performance characteristics of the two assay formats under identical experimental conditions.

A key limitation of this study is the small sample size, particularly with respect to ROC-based analyses. Although ROC curves do not require continuous predictors, the presence of only two ELISA-positive samples limits the ability to derive statistically robust estimates of sensitivity or specificity. Accordingly, ROC analysis in this study should be interpreted as a descriptive visualization of assay alignment rather than a formal assessment of diagnostic performance. Future studies with larger sample sizes are required to validate these observations quantitatively.

### Future directions

To make lateral flow assays more viable for neural tissue proteins, one could explore signal amplification strategies on the strip. For instance, using larger gold nanoparticles or silver enhancement on the gold particles can improve visibility. Another approach would be to develop a reader that detects earlier color development or uses fluorescence to quantify line intensity. With appropriate calibration, a quantitative LFA reader could report an approximate concentration (e.g., “AChE ~20 ng/mL”) which would narrow the gap with ELISA. Additionally, multiplexing the strip to detect multiple neural proteins simultaneously could provide broader diagnostic information (e.g., an array of lines for different enzymes or structural proteins). However, multiplexing can further reduce sensitivity if not carefully designed. From a sample prep perspective, integrating a small tissue homogenizer or microfluidic device to extract the protein and feed it into a lateral flow strip could create a true point-of-care device for tissue analysis. These remain engineering challenges. For now, in standard lab practice, one might use lateral flow as a quick screening and follow up with ELISA (or another quantitative method) for confirmation and detailed analysis. In context of neuropathology, AChE staining is often done histochemically on tissue sections to visualize nerve fibers. Our study suggests that soluble AChE can also be quantified biochemically even after fixation. This could open up complementary approaches: for instance, measuring AChE biochemically to supplement histology in autopsy samples of peripheral neuropathy or in evaluating nerve graft viability. The choice of method will depend on required sensitivity and infrastructure. If only a small portable kit is available (e.g., in a field lab), the colloidal gold test might provide a quick answer to whether AChE is present above a threshold. If precise measurement or detection of very low levels is needed (for example, early degeneration where AChE drops slightly), an ELISA or other lab method is necessary.

## Conclusion

We evaluated ELISA and colloidal gold lateral flow strip methods for detecting AChE protein in formalin-fixed cadaveric nerve tissues. The ELISA proved to be far more sensitive, detecting AChE in all samples including those with very low levels, and provided quantitative results with high reproducibility. The colloidal gold strip, in contrast, only identified AChE in samples where the concentration was above ~10 ng/mL, acting as a qualitative threshold test. It failed to detect lower concentrations that ELISA picked up, and it did not offer precise quantification. Bland–Altman analysis confirmed that the two methods are not interchangeable: the lateral flow assay systematically underestimated AChE content relative to ELISA. In practical terms, ELISA remains the gold standard for accurate measurement of neural protein biomarkers, especially in scenarios requiring sensitivity (e.g., research measurements of enzyme levels or subtle diagnostic changes). Colloidal gold lateral flow tests, while rapid and convenient, should be used with caution for neural tissue diagnostics, they are suitable for quick yes/no answers when protein levels are high, but negative results must be interpreted in light of their higher detection limit. Future improvements in lateral flow technology or the use of reader devices may narrow this gap. Until then, critical assessments of neural proteins, particularly from fixed or limited samples, are best served by lab-based immunoassays. Our study provides a reference point for the performance one can expect from these two assay formats and underscores the importance of choosing the right tool for the analytical task at hand.

## Data Availability

The original contributions presented in the study are included in the article/supplementary material, further inquiries can be directed to the corresponding author.
